# In Vivo Cerebral Translocator Protein (TSPO) Binding and Its Relationship with Blood Adiponectin Levels in Treatment-Naïve Young Adults with Major Depression: A [^11^C]PK11195 PET Study

**DOI:** 10.3390/biomedicines10010034

**Published:** 2021-12-24

**Authors:** Yo-Han Joo, Min-Woo Lee, Young-Don Son, Keun-A Chang, Maqsood Yaqub, Hang-Keun Kim, Paul Cumming, Jong-Hoon Kim

**Affiliations:** 1 Neuroscience Research Institute, Gachon University, Incheon 21565, Korea; yhjoo@gachon.ac.kr (Y.-H.J.); minwoo1125@medicalip.com (M.-W.L.); ydson@gachon.ac.kr (Y.-D.S.); keuna705@gachon.ac.kr (K.-A.C.); dsaint@gachon.ac.kr (H.-K.K.); 2 Department of Biomedical Engineering, College of Health Science, Gachon University, Incheon 21936, Korea; 3 Gachon Advanced Institute for Health Science and Technology, Graduate School, Gachon University, Incheon 21565, Korea; 4 Department of Pharmacology, Gachon University College of Medicine, Gachon University, Incheon 21936, Korea; 5 Department of Radiology and Nuclear Medicine, Amsterdam University Medical Centers, 1081 HV Amsterdam, The Netherlands; maqsood.yaqub@amsterdamumc.nl; 6 Department of Nuclear Medicine, Inselspital, Bern University, CH-3010 Bern, Switzerland; paul.cumming@insel.ch; 7 School of Psychology and Counselling, Queensland University of Technology, Brisbane 4059, Australia; 8 Department of Psychiatry, Gachon University College of Medicine, Gil Medical Center, Gachon University, Incheon 21565, Korea

**Keywords:** translocator protein, positron emission tomography, [^11^C]PK11195, adiponectin, major depression

## Abstract

Adiponectin is an adipokine that mediates cellular cholesterol efflux and plays important roles in neuroinflammatory processes. In this study, we undertook positron emission tomography (PET) with the translocator protein (TSPO) ligand [^11^C]PK11195 and measured serum adiponectin levels in groups of treatment-naïve young adult patients with major depressive disorder (MDD) and matched healthy controls. Thirty treatment-naïve MDD patients (median age: 24 years) and twenty-three healthy controls underwent [^11^C]PK11195 PET. We quantified TSPO availability in brain as the [^11^C]PK11195 binding potential (BP_ND_) using a reference tissue model in conjunction with the supervised cluster analysis (SVCA4) algorithm. Age, sex distribution, body mass index, and serum adiponectin levels did not differ between the groups. Between-group analysis using a region-of-interest approach showed significantly higher [^11^C]PK11195 BP_ND_ in the left anterior and right posterior cingulate cortices in MDD patients than in controls. Serum adiponectin levels had significant negative correlations with [^11^C]PK11195 BP_ND_ in the bilateral hippocampus in MDD patients, but significant positive correlations in the bilateral hippocampus in the control group. Our results indicate significantly higher TSPO binding in the anterior and posterior cingulate cortices in treatment-naïve young MDD patients, suggesting microglial activation in these limbic regions, which are involved in cognitive and emotional processing. The opposite correlations between [^11^C]PK11195 BP_ND_ in the hippocampus with serum adiponectin levels in MDD and control groups suggest that microglial activation in the hippocampus may respond differentially to adiponectin signaling in MDD and healthy subjects, possibly with respect to microglial phenotype.

## 1. Introduction

The high prevalence of major depressive disorder (MDD) in patients with chronic inflammatory illnesses [1,2,3] suggests a causal association between MDD and inflammatory processes. Indeed, a subset of patients with MDD showed altered cytokine levels in the blood, which were associated with antidepressant treatment responses [4]. Furthermore, a recent meta-analysis provides evidence that the levels of inflammatory markers, such as pro-inflammatory cytokines and chemokines, are significantly elevated in the cerebrospinal fluid (CSF) of patients with MDD [5].

Positron emission tomography (PET) imaging with ligands for the 18-kDa translocator protein (TSPO) enables the assessment in the living human brain of microglial activation, which is an index of the state of neuroinflammation in vivo [6,7]. So far, several PET studies with first- or second-generation tracers have reported on cerebral TSPO binding in patients with MDD [8,9,10,11,12,13,14,15,16]. Among these studies, most reported elevated TSPO binding in MDD in various cerebral regions, including the anterior cingulate cortex, posterior cingulate cortex, prefrontal cortex, insula, hippocampus, and temporal cortex [9,10,11,13,14,15]. One PET study found no differences in TSPO availability between MDD and healthy control subjects [8]. While the cellular localization of the TSPO signal is not always certain, the near consensus of PET results is consistent with microglial activation or monocyte recruitment in brain of MDD patients [5,17].

Previous TSPO PET studies of MDD used patient groups that were inhomogeneous with respect to age and medication history. Confounding factors such as previous exposure to antidepressants, duration of antidepressant use, body mass index, and age can all affect TSPO binding [13,15,18,19]. In addition, TSPO expression may change as a function of disease progression in patients with MDD [15], which might speak to the clinical importance of modulating and reducing microglial activation in early stages of the illness [15,20].

Recent attempts to find a relationship between peripheral blood markers and cerebral TSPO binding in MDD have not yielded successful results [5]. It is important to find a practical and applicable peripheral measure associated with brain microglial activation [21]. In the present study, we focused on the relationship between blood levels of the anti-inflammatory molecule adiponectin and cerebral TSPO binding levels. Adiponectin is an emerging peripheral biomarker of MDD [22], which has been shown by meta-analysis to be lower in patients with MDD than in healthy controls [23]. A 30 kDa peptide secreted by adipocytes, adiponectin exerts its anti-inflammatory effects via transmembrane receptors [22]. In addition to its roles in the regulation of lipid and glucose metabolism, adiponectin has central effects on brain homeostasis, neuroprotection, cognition, affect, and reward [24]. In particular, adiponectin receptors are widely expressed in the brain, including the prefrontal cortex and hippocampus, implying an important role in affect and cognition [25]. Most importantly, previous preclinical studies have reported that adiponectin receptors regulate the polarization and function of microglia through downstream effects on peroxisome proliferator-activated receptor (PPAR)-γ and AMP-activated protein kinase (AMPK) signaling pathways [26,27,28], suggesting that adiponectin plays a crucial role in modulating microglial activity.

Accumulating evidence also suggests that adiponectin has neurotrophic properties and promotes synaptic plasticity in the hippocampus, manifesting antidepressant-like behavioral effects in rodents [29]. Adiponectin stimulates the proliferation of adult hippocampal neural stem cells in vitro by acting on mitogen-activated protein kinase (MAPK) and glycogen synthase kinase 3β (GSK3β) pathways [30]. Lower baseline blood adiponectin levels are also associated with the rapid antidepressant response to ketamine treatment, which has suppressive effects on neuroinflammation in MDD [31]. These preclinical and clinical observations point to a potential link between blood adiponectin and TSPO expressed on activated microglia in MDD patients.

Therefore, we aimed in this study to examine cerebral TSPO binding using [^11^C]PK11195 PET and measure blood adiponectin levels in homogeneous groups of treatment-naïve young adult patients with MDD and matched healthy controls. Our goal was to examine the increased TSPO binding in our cohort of treatment-naïve young MDD patients and to explore the relationship between the individual blood levels of adiponectin and regional TSPO binding in brain of patients and controls.

## 2. Materials and Methods

### 2.1. Participants

The Institutional Review Board of the Gachon University Gil Medical Center approved the study protocol, which we conducted in accordance with international ethical standards and the Declaration of Helsinki. All participants gave written informed consent after they had received a full explanation of the study procedures. Our study aimed to measure cerebral TSPO availability in a homogeneous group of treatment-naïve young adult patients with MDD. As such, the first inclusion criterion was (i) age from 20 to 35 years. Other inclusion criteria were (ii) diagnosis of MDD according to the Diagnostic and Statistical Manual of Mental Disorders 4th edition (DSM-IV) [32], which was established by the Structured Clinical Interview for DSM-IV (SCID-IV) [33], with no other current or previous Axis I diagnosis. Patients with comorbid anxiety disorders such as generalized anxiety disorder, panic disorder, phobic disorders, obsessive-compulsive disorder, or posttraumatic stress disorder were excluded; (iii) no past or current substance abuse/dependence; (iv) no history of medical or neurological disorders; (v) no past or current history of psychiatric treatments; and (vi) no past or current use of any psychotropic medications (such as antidepressants, benzodiazepines/anxiolytics, hypnotics, antipsychotics, or mood stabilizers). In this study, we enrolled patients with MDD diagnosed by the DSM-IV instead of DSM-5. The core symptoms applied to the diagnosis of MDD and the requisite duration of at least two weeks did not change from DSM-IV to DSM-5 [34]. In DSM-5, the coexistence of manic symptoms within an MDD episode, which are insufficient to satisfy the criteria for a manic episode, qualifies as “mixed features”. This inclusion of mixed features in MDD as diagnosed by DSM-5 increases the likelihood of recruiting MDD patients with a bipolar spectrum. Since we focused our study on a homogeneous group of patients with pure MDD, we therefore used the DSM-IV. In addition, we decided to be consistent with previous TSPO PET studies on MDD, in which DSM-IV criteria were used. We enrolled 30 patients meeting these criteria (Table 1). The patients’ mean age was 24.6 ± 4.2 (median: 24) years and mean duration of current episode of depression was 1.7 ± 1.0 months. We also recruited a group of 23 healthy control subjects, who met the criteria of no past or current psychiatric, neurological, or medical disorders, and no past or current use of medications affecting the central nervous system, and obtained their written informed consent to undergo the scanning protocols. None of the participants showed any brain structural abnormalities on magnetic resonance imaging (MRI), as confirmed by a board-certified radiologist. Patients with MDD were recruited from outpatient clinics and through local advertisements, and control subjects were recruited through local advertisements. We did not perform a sample size estimation before the subject recruitment. However, the number of our sample (30 treatment-naïve MDD patients and 23 healthy controls) is clearly higher than or comparable to that of previous TSPO PET studies in MDD. In addition, this sample size is generally considered adequate for a radioligand PET study of drug-naïve psychiatric patients [35].

### 2.2. Clinical Assessments

Clinical assessments were conducted using the Hamilton Rating Scale for Depression (HAMD-17) [36], Beck Depression Inventory (BDI) [37], Rosenberg Self-Esteem Scale (RSES) [38], and Barratt Impulsiveness Scale (BIS) [39]. For the HAMD-17, BDI, and BIS, higher scores indicate more severe symptoms, whereas higher RSES scores indicate higher self-esteem.

### 2.3. Scanning Protocol for [^11^C]PK11195 PET Imaging

All participants were scanned using a Biograph 6 PET scanner (Siemens Medical Imaging Systems, Knoxville, TN, USA) with ^11^C-(*R*)-[1-(2-chlorophenyl)-*N*-methyl-*N*-(1-methyl-propyl)-3-isoquinoline-carboxamide] ([^11^C]PK11195). Following bolus injection of a mean dose of 640 ± 58 MBq [^11^C]PK11195 with a mean molar activity of 44 ± 15 GBq/μmol, a dynamic emission recording lasting 60 min was initiated in list mode. A computed tomography (CT)-based transmission scan was performed immediately before the tracer injection and used for attenuation correction of the PET data. The [^11^C]PK11195 PET images were reconstructed using the two-dimensional ordered-subset expectation maximization (OSEM-2D) algorithm. The reconstructed PET images had a matrix size of 256 × 256 × 109 and a voxel size of 1.33 × 1.33 × 1.50 mm^3^. To calculate the [^11^C]PK11195 binding potential with respect to non-displaceable compartment (BP_ND_), the emission data of [^11^C]PK11195 PET were reconstructed into 21 frames of the following duration: 2 × 15 s, 3 × 30 s, 3 × 60 s, 2 × 90 s, 3 × 120 s, 2 × 180 s, 4 × 300 s, and 2 × 600 s (60 min in total). Attenuation, scatter, and decay time correction were applied for each frame.

For normalization of the PET images to the Montreal Neurological Institute (MNI) space, 3-Tesla MRI (Magnetom Verio; Siemense, Erlangen, Germany) scans were performed using a 3-dimensional T1-weighted magnetization prepared rapid gradient echo (3-D T1MPRAGE) sequence for structural brain imaging. The 3-D T1MPRAGE images were acquired with the following parameters: repetition time = 1900 ms, echo time = 3.3 ms, inversion time = 900 ms, flip angle = 9°, voxel size = 0.5 × 0.5 × 1.0 mm^3^, matrix size = 416 × 512, and number of slices = 160.

### 2.4. [^11^C]PK11195 PET Imaging Analysis

**Figure 1:**
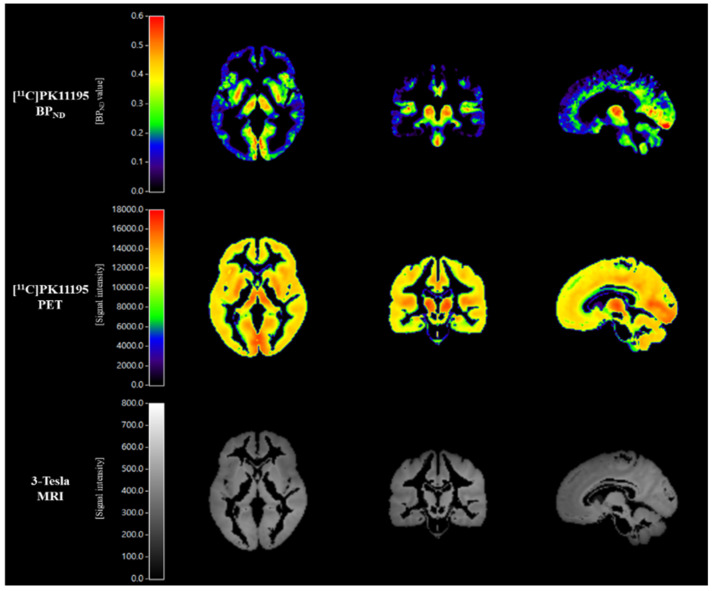
Representative mean images of [^11^C]PK11195 BP_ND_, [^11^C]PK11195 PET, and corresponding 3-Tesla MRI in the control group.

The 3-D T1MPRAGE MRI images of each subject were co-registered to their corresponding PET summation images using Statistical Parametric Mapping 12 (SPM12; Wellcome Trust Center for Neuroimaging, London, UK). The co-registered MRI images were spatially normalized to the MNI template using SPM12 with a nonlinear deformation field, and the estimated transform was applied to the corresponding resampled PET images. To extract reference regions of [^11^C]PK11195 PET images, we used the supervised cluster analysis approach [40,41,42]. The modified supervised cluster analysis (SVCA4) algorithm was applied to identify brain regions with [^11^C]PK11195 binding of four kinetic classes: (1) gray matter with specific [^11^C]PK11195 binding, (2) gray matter without specific binding, (3) white matter, and (4) blood pool [42]. The SVCA4 analysis assigns each voxel in the masked [^11^C]PK11195 PET images into one of the four classes based on their time-activity curves [42,43]. Using the gray matter clusters without specific binding as a reference region, the [^11^C]PK11195 BP_ND_ images were calculated by receptor parametric mapping 2 (RPM2), which is a basis function implementation of the simplified reference tissue model 2 (SRTM2) [44]. Representative examples of [^11^C]PK11195 PET and 3-Tesla MR images are shown in Figure 1.

We calculated regional BP_ND_ values in the a priori regions of interest (ROIs), i.e., the prefrontal cortex, anterior and posterior cingulate cortices, insula, hippocampus, and temporal cortex. We selected these ROIs based on earlier reports of elevated TSPO binding in patients with MDD. The anatomical location of the prefrontal cortex ROI was determined based on the Brodmann areas in the Talairach atlas [45], which includes the dorsolateral, medial, ventrolateral, and orbitofrontal subregions [46]. We defined the remaining a priori ROIs using the automated anatomical labeling (AAL) program [47]. The left and right regions of a priori ROIs were analyzed separately, since previous studies have reported functional imbalance between the left and right hemispheric regions in depression [48,49].

### 2.5. Measurement of Blood Adiponectin Levels

Measurement of blood adiponectin was performed on the same day of the PET scan prior to the scan. Venous blood samples were collected from participants after at least four hours of fasting. Blood samples were collected in SST tubes (BD367954, Fisher Scientific, Loughborough, UK) and centrifuged at 3000 rpm for 10 min at 4 °C. After separating the serum, protease inhibitor cocktail (535140; EMD Biosciences, Inc., Darmstadt, Germany), and phosphatase inhibitor cocktail (P5726 and P0044; Sigma-Aldrich, Inc., St. Louis, MO, USA) were added, and samples were immediately stored at −80 °C until analysis. After thawing, serum samples were diluted 200-fold, and adiponectin levels were analyzed using an enzyme-linked immunosorbent assay (ELISA) kit (DRP300, R&D systems, Minneapolis, MN, USA) following the manufacturer’s instructions, with calculation of the mean of duplicate values for each case. Quantification of adiponectin protein levels was detected using a VICTOR X4 Multimode Plate Reader (PerkinElmer, Waltham, MA, USA). Serum adiponectin levels were successfully measured in 26 of 30 patients with MDD and in 21 of 23 control subjects. In four patients and two controls, the levels were not available due to hemolysis. In the analysis of TSPO availability and adiponectin levels, the investigators were blinded to group allocation.

### 2.6. Statistical Analysis

The between-group comparisons of the [^11^C]PK11195 BP_ND_ values of *a priori* ROIs were performed using two-sample *t*-tests. Since we aimed to examine the TSPO binding in a priori ROIs in which elevated TSPO binding was reported in MDD patients, we determined the level of statistical significance at a two-tailed *p* < 0.05. The relationship between clinical severity and [^11^C]PK11195 BP_ND_ of ROIs was examined using Pearson’s correlation analysis. The association between serum adiponectin levels and [^11^C]PK11195 BP_ND_ values of ROIs was evaluated using Pearson’s correlation analysis. Since there were no previous reports on the relationship between blood adiponectin levels and cerebral TSPO binding in treatment-naïve young patients with MDD, the level of statistical significance on the correlation between adiponectin levels and regional cerebral TSPO binding was set at a two-tailed *p* < 0.05 without the correction for multiple correlations.

## 3. Results

The demographic and clinical characteristics of the participants are presented in Table 1. Age, sex distribution, body mass index (BMI), serum adiponectin levels, and scan parameters did not differ between the groups (Table 1). The patients’ mean HAMD-17 score was 24.3 ± 6.7 (Table 1). The mean BDI and BIS scores were significantly higher in MDD patients than in controls, while the score of self-esteem measured by the RSES was significantly higher in controls than in MDD patients (*p* < 0.0001) (Table 1).

**Figure 2:**
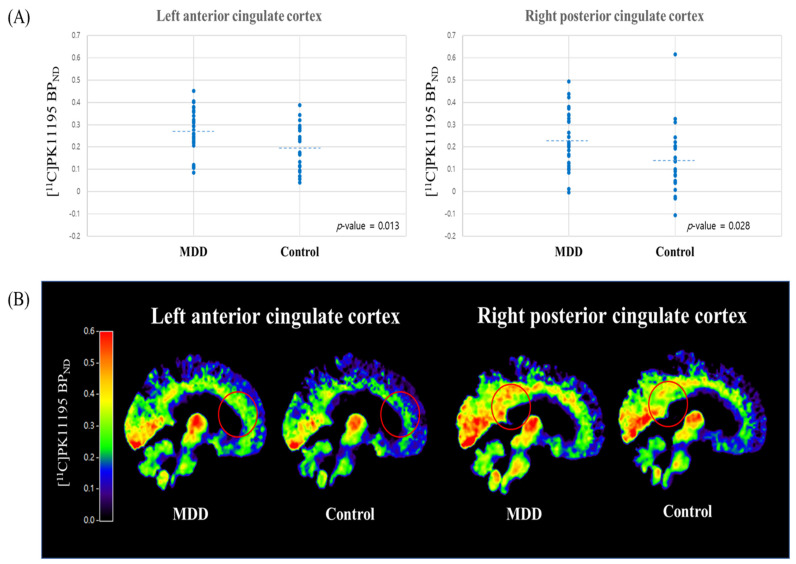
(**A**) Distribution of [^11^C]PK11195 BP_ND_ values in the left anterior cingulate and right posterior cingulate cortices in MDD patients (*n* = 30) and controls (*n* = 23). The dashed lines indicate the average values of each distribution. The [^11^C]PK11195 BP_ND_ values were significantly higher in the left anterior (*p* = 0.013) and right posterior cingulate (*p* = 0.028) cortices in MDD patients than that in control subjects. (**B**) Mean [^11^C]PK11195 BP_ND_ images in a representative sagittal plane in MDD patients (*n* = 30) and controls (*n* = 23). Region of interest analysis indicated significantly higher [^11^C]PK11195 BP_ND_ in the left anterior and right posterior cingulate cortices (marked by red circles) in MDD patients than in control subjects. BP_ND_, binding potential with respect to non-displaceable compartment; MDD; major depressive disorder.

The ROI-based between-group analysis showed significantly higher [^11^C]PK11195 BP_ND_ in the left anterior cingulate cortex (effect size = 0.713, *p* = 0.013) and right posterior cingulate cortex (effect size = 0.629, *p* = 0.028) in MDD patients than in controls (Table 2; Figure 2). Patients with MDD showed a non-significant tendency towards higher [^11^C]PK11195 BP_ND_ in the left prefrontal cortex (effect size = 0.494, *p* = 0.080) compared to controls (Table 2).

**Figure 3:**
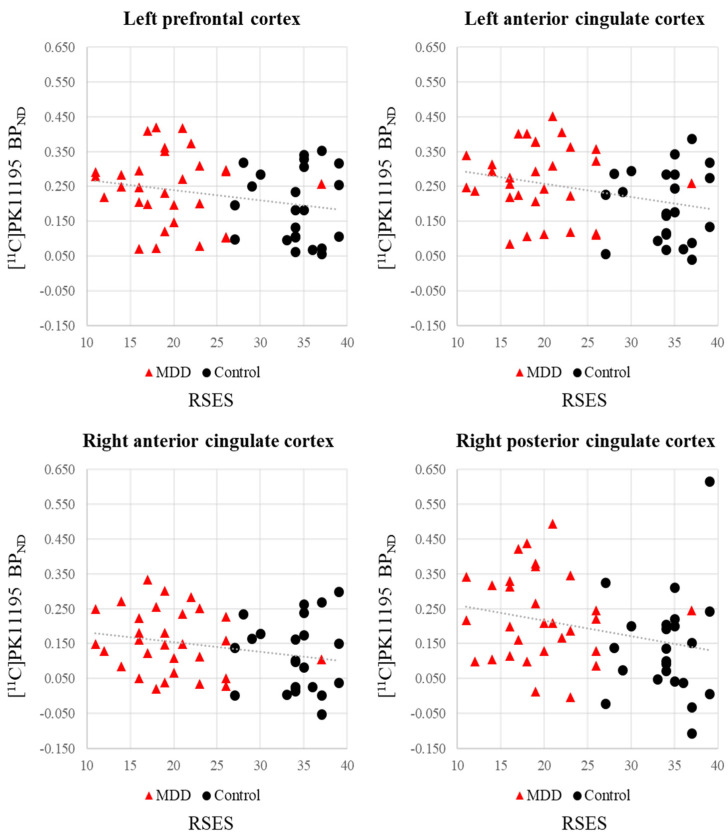
Scatter plots of the correlation between [^11^C]PK11195 BP_ND_ values and RSES scores for the entire subject group (*n* = 53). The RSES score had a significant negative correlation with the [^11^C]PK11195 BP_ND_ in the left anterior cingulate cortex (*r* = −0.301, *p* = 0.029) and tended to have negative correlations with the [^11^C]PK11195 BP_ND_ in the left prefrontal (*r* = −0.242, *p* = 0.081), right anterior cingulate (*r* = −0.248, *p* = 0.074), and right posterior cingulate (*r* = −0.269, *p* = 0.052) cortices. BP_ND_, binding potential with respect to non-displaceable compartment; MDD, major depressive disorder; RSES, Rosenberg Self-Esteem Scale.

In the patient group, [^11^C]PK11195 BP_ND_ values did not significantly correlate with the HAMD-17, BDI, BIS, or RSES scores (*p* > 0.05) (Supplementary Table S1). In the entire subject group (patients and controls), Pearson’s correlation analysis showed a significant negative correlation between the [^11^C]PK11195 BP_ND_ in the left anterior cingulate cortex and the RSES score (*r* = −0.301, *p* = 0.029) (Table 3; Figure 3). The RSES score also tended to have negative correlations with [^11^C]PK11195 BP_ND_ in the left prefrontal cortex (*r* = −0.242, *p* = 0.081), the right anterior cingulate cortex (*r* = −0.248, *p* = 0.074), and the right posterior cingulate cortex (*r* = −0.269, *p* = 0.052) (Table 3; Figure 3).

**Figure 4:**
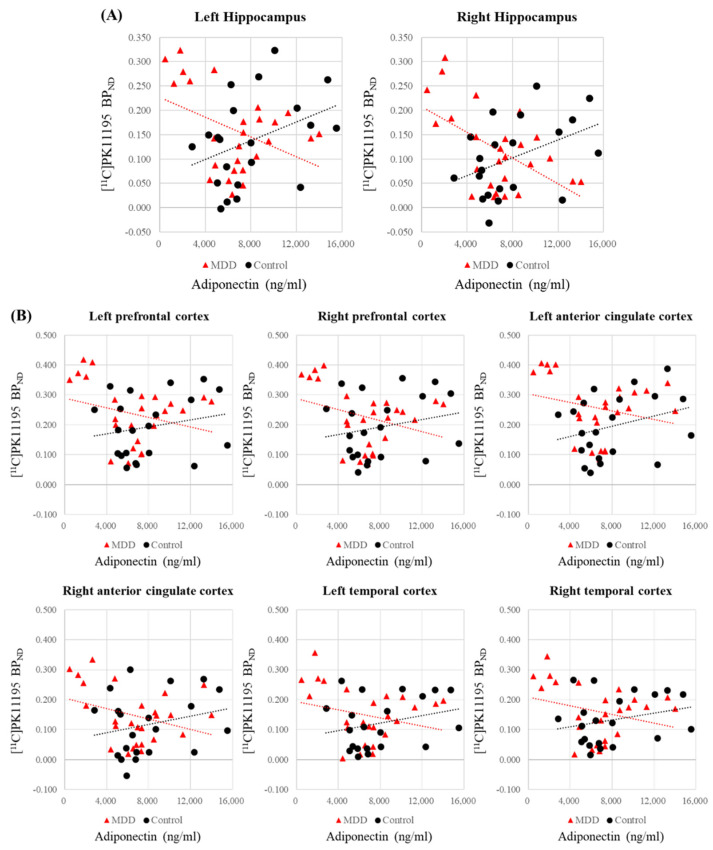
(**A**) Scatter plots of the correlation between serum adiponectin levels and [^11^C]PK11195 BP_ND_ values in the hippocampus for the MDD and control groups. The Fisher’s Z-transformation analysis revealed significant between-group differences in correlation coefficients (left hippocampus: *z* score = −3.3, *p* < 0.001; right hippocampus: *z* score = −4.2, *p* < 0.001). (**B**) Scatter plots of the correlation between serum adiponectin levels and [^11^C]PK11195 BP_ND_ values in the bilateral prefrontal, anterior cingulate, and temporal cortices. The Fisher’s Z-transformation analysis revealed significant between-group differences in correlation coefficients (*p* < 0.05), indicating negative correlations in the MDD group and positive correlations in the control group. BP_ND_, binding potential with respect to non-displaceable compartment; MDD, major depressive disorder.

The correlations between serum adiponectin levels and regional [^11^C]PK11195 BP_ND_ are presented in Table 4. In the MDD group, [^11^C]PK11195 BP_ND_ in all ROIs showed negative correlations with serum adiponectin levels, while the control group showed positive correlations in all ROIs (Table 4). The correlation analysis in the MDD group with BMI as a covariate showed significant negative correlations between the serum adiponectin levels and the [^11^C]PK11195 BP_ND_ values in the left hippocampus (*r* = −0.433, *p* = 0.030) and right hippocampus (*r* = −0.564, *p* = 0.003) (Table 4; Figure 4A). The correlation analysis in the control group with BMI as a covariate showed significant positive correlations between the serum adiponectin levels and the bilateral hippocampal [^11^C]PK11195 BP_ND_ values (left: *r* = 0.520, *p* = 0.019; right: *r* = 0.594, *p* = 0.006) (Table 4; Figure 4A). The Fisher’s Z-transformation analysis revealed significant between-group differences in correlation coefficients between the serum adiponectin levels and the [^11^C]PK11195 BP_ND_ values in the left hippocampus (*z* = −3.305, *p* = 0.001) and right hippocampus (*z* = −4.200, *p* < 0.001) (Table 4; Figure 4A). The Fisher’s Z-transformation analysis also showed significant between-group differences in correlation coefficients between the serum adiponectin levels and the [^11^C]PK11195 BP_ND_ values in the bilateral prefrontal (*z* score: left: −2.134; right: −2.330), anterior cingulate (*z* score: left: −2.320, right: −2.381), and temporal (*z* score: left: −2.256, right: −2.384) cortices (*p* < 0.05) (Table 4; Figure 4B), consistently indicating negative correlations in the MDD patients and positive correlations in the control subjects.

## 4. Discussion

Our analysis of [^11^C]PK11195 PET data showed significantly higher [^11^C]PK11195 BP_ND_ in the anterior and posterior cingulate cortices in a group of 30 treatment-naïve MDD patients than in the control group, corresponding to Cohen’s d effect sizes of 0.63 and 0.71, respectively. This result is in good agreement with previous PET studies using various TSPO ligands in groups of MDD patients who were heterogeneous with respect of age and antidepressant treatment history [9,10,11,13,14,15]. A recent multicenter study called for replication of PET neuroimaging findings across groups of adequate sample size [50]. This is particularly important in the field of psychiatric disorders, which are associated with complex clinical pictures and the absence of clear objective diagnostic tools. Thus, increased abundance of TSPO binding sites emerges as one of the most consistent biomarkers for MDD, comparable in effect size to the relative reductions in serotonin transporter availability [51].

We also found that the availability of TSPO binding sites in the hippocampus of patients with MDD had a significant negative correlation with serum adiponectin levels; the correlation analysis indicated that adiponectin accounted for about 10% of the variance of BP_ND_ in the population, as compared to a total variance of about 50%. Overall, these results indicate higher in vivo cerebral TSPO binding in treatment-naïve young adult patients with MDD, and an inverse relationship between cerebral TSPO binding and serum adiponectin in these patients. The relationship patterns between the two measures were significantly different between the MDD and control groups. To our knowledge, this is the first report on TSPO binding in a homogeneous group of treatment-naïve young adults with MDD and is the first combined study of TSPO PET and serum levels of an anti-inflammatory adipokine.

In our study, we chose a priori ROIs (prefrontal cortex, anterior and posterior cingulate cortices, insula, hippocampus, and temporal cortex) based on previous PET studies measuring TSPO binding in MDD, in which patients differed in terms of treatment status and age. Our study using a highly homogeneous cohort of treatment-naïve young adults supports the previous PET studies in which elevated TSPO binding in the anterior cingulate cortex was a consistent finding in MDD [5]. In addition, we found that [^11^C]PK11195 BP_ND_ was significantly higher in the posterior cingulate cortex in the patient group than in the control group. Present findings of increased TSPO binding in the cingulate cortex of MDD patients may relate to a disruption of top-down cortical network processing, particularly of the default mode network [20,52]. Since the anterior cingulate cortex is critically involved in the modulation of affective inputs from ventral stream areas [53,54], our results suggest that neuroinflammatory reactions in the cingulate cortex may contribute to perturbations of bottom-up and top-down processing in MDD. We note that this pattern of increased TSPO binding in the cingulate cortex overlaps with cerebral blood flow disturbances in patients with depression [55]. Multimodal imaging might establish better the relationship between TSPO expression and functional indices such as cerebral perfusion rate or cerebral metabolism.

The level of self-esteem measured using the RSES had significant negative correlations with regional [^11^C]PK11195 BP_ND_ across the entire subject group. As shown in Figure 4, the RSES score lies along a continuum from 11 to 39, thus representing a dimensional rather than a categorical distribution. Low self-esteem represents a core symptom of MDD and is a significant risk factor for depression in the general population [56]. Low self-esteem also brings a higher suicide risk in psychiatric patients, particularly those with MDD [57]. In addition, it was reported that low self-esteem was associated with suicide intent independently of the severity of depressive symptoms [58]. Previous PET studies reported that severity of depression and presence of suicidal ideation had associations with elevated TSPO binding in MDD patients [10,14]. Future studies including a larger population of patients and control subjects expressing a greater range of self-esteem scores might confirm this relationship. Furthermore, our findings may support the notion that inflammatory responses in brain regions such as parts of the prefrontal cortex and anterior cingulate are associated with sickness behaviors that overlap with depressive symptoms [59,60].

Adiponectin has a potent role in the reduction in oxidative stress and inhibition of inflammation, in addition to its actions in energy and glucose homeostasis in peripheral tissues [22]. Moreover, adiponectin crosses the blood–brain barrier and plays a prominent role in neuroprotection and neurogenesis in the hippocampus, where adiponectin receptors are highly expressed [61,62,63]. In a rodent study, intracerebroventricular injection of an adiponectin antibody provoked depression-like behaviors, while administration of adiponectin into the brain produced antidepressant-like effects [61].

It is notable that the TSPO binding in the bilateral hippocampus showed a negative correlation with serum adiponectin levels in the patient group (left; *r* = −0.43: right; *r* = −0.56), while the reverse relationship was observed in healthy controls (left; *r* = 0.52: right; *r* = 0.59). Thus, the normal relationship between serum adiponectin and TSPO availability is reversed in the condition of MDD. In addition, there were also significant between-group differences in correlation coefficients between the serum adiponectin levels and the [^11^C]PK11195 BP_ND_ values in other ROIs including the bilateral prefrontal, anterior cingulate, and temporal cortices, consistently indicating negative correlations in the MDD group and positive correlations in the control groups. In our study, we used BMI as a covariate since BMI had a weak inverse relationship with blood adiponectin levels [64] and TSPO binding [19]. In any event, BMI cannot be a factor in our study, since BMI was matched between the groups (23.6 ± 4.1 vs. 23.6 ± 3.3, *p* = 0.998). Previous studies reported a general lack of correlations between central and peripheral inflammatory biomarkers in MDD [14,65]. However, a recent PET report showed significant positive correlations between the brain distribution volume (V_T_; mL g^−1^) of a TSPO tracer and serum levels of selected inflammatory markers, i.e., prostaglandin E2 and tumor necrosis factor alpha, in patients with MDD [21]. As such, these and other peripheral inflammatory markers may be associated with increased TSPO availability in the brain of MDD patients; although, the direction of causality is a matter of conjecture. In our study, adiponectin levels were non-significantly lower in patients with MDD, but the correlation analysis implies that serum adiponectin enhances TSPO expression in healthy controls while reducing TSPO expression in MDD patients. Our findings of significant inverse correlations in MDD could be interpreted as a phenomenon where reduced adiponectin and thereby diminished anti-inflammatory properties predispose to increased inflammatory responses in this critical limbic region in MDD patients. Invoking the bivalent nature of TSPO binding with respect to the phenotype of microglia may also help to interpret a differential pattern of relationships. Microglia can assume the pro-inflammatory (M1) and anti-inflammatory (M2) phenotypes [66], which are indistinguishable by TSPO PET ligands, such that findings of increased TSPO in MDD are formally ambiguous with respect to microglial function. In vitro studies suggest that pharmacological activation of TSPO can modulate the differentiation of microglia between anti-inflammatory and pro-inflammatory microglial phenotypes [67]. We suppose that MDD may entail a reversal of the phenotypic expression of microglia in relation to adiponectin levels. Adiponectin might promote expression of TSPO in microglia of an anti-inflammatory phenotype in healthy controls, thus accounting for the positive correlation, while favoring a pro-inflammatory phenotype in patients with MDD. Our results are in line with previous reports that the action of adiponectin is state-dependent and multifaceted [24]. Since the activation states and phenotypes of microglia are complex and dynamic depending on the cellular environments and endogenous hormonal signaling in healthy and diseased brains [68], our results suggest that adiponectin may be a blood biomarker that is differentially associated with TSPO levels expressed on microglia in MDD patients and healthy subjects. In addition, we note that TSPO binding in neuroinflammatory states mainly reflects a varying degree of microglial activation, whereas TSPO binding in healthy brain may be confined largely to endothelial cells [69,70]. Furthermore, the cellular sources and functional significance of TSPO expression may differ in healthy and diseased brains [70,71].

We note some limitations in the interpretation of the present results. By design, all patients were treatment-naïve young adults, such that findings might not be generalizable to a more heterogeneous group of MDD patients. We quantified TSPO availability using the supervised cluster analysis approach [40,42], which has been implemented in other TSPO PET studies in MDD [9,10]. With some caveats, this approach is preferable to traditional methods where the cerebellum or white matter is used as a reference region. However, the BP_ND_ values are still vulnerable to bias from uncorrected contamination of the reference cluster by specific binding [7]. Since there is no brain region completely devoid of TSPO [72], the gold standard compartmental analysis using the metabolite-corrected arterial input function might have been preferable, had the invasive procedure been available to us. We did not evaluate lifestyle factors such as diet, exercise, and physical activity that can affect microglial functioning and aberrant neuroinflammation [73,74]. Future studies should assess these factors to address the relationships among confounding lifestyle factors, neuroinflammation, and peripheral markers in MDD. In this study, we did not aim to correlate conventional blood markers of inflammation such as cytokines with cerebral TSPO binding, since previous studies have found no significant correlations (for review, refer to [5]). However, further studies are clearly required to measure novel peripheral cytokines and chemokines to unravel the complex relationships between peripheral and central markers of inflammation in healthy and diseased brains.

## 5. Conclusions

Our study provides evidence of significantly higher in vivo cerebral TSPO binding in the anterior and posterior cingulate cortices in treatment-naïve young adult patients with MDD, which may indicate neuroinflammation in these important limbic cortical regions mediating bottom-up and top-down processing. The TSPO binding level in the bilateral hippocampus had significant inverse correlations with serum adiponectin levels in MDD, whereas there were significant positive correlations in the healthy control group, suggesting that adiponectin signaling, which is considered one of the endogenous factors that regulate microglial activation and phenotypes in the brain, may play different roles in MDD patients and healthy subjects.

## Figures and Tables

**Table 1:** Demographic/clinical characteristics and PET scan parameters.

Variables	MDD (*n* = 30)	Controls (*n* = 23)	*t*-Value	*p*-Value
Age (year)	24.6 ± 4.2	24.5 ± 3.2	0.074	0.941
Gender (male/female)	13/17	13/10	0.906 (χ^2^)	0.341
Duration of current episode (months)	1.7 ± 1.0	-	-	-
HAMD-17	24.3 ± 6.7	-	-	-
BDI	27.2 ± 9.0	1.5 ± 1.7	15.292	<0.0001
RSES	19.5 ± 5.4	34.0 ± 3.6	−11.053	<0.0001
BIS	72.2 ± 10.5	58.1 ± 9.4	5.066	<0.0001
BMI	23.6 ± 4.1	23.6 ± 3.3	−0.002	0.998
Adiponectin (ng/mL)	6668 ± 3451	8024 ± 3597	−1.314	0.196
Injected dose (MBq)	631.5 ± 60.9	651.6 ± 52.5	−1.264	0.212
Specific activity (GBq/umol)	43 ± 12	44 ± 16	−0.299	0.767

PET, positron emission tomography; MDD, major depressive disorder; HAMD-17, Hamilton Rating Scale for Depression; BDI, Beck Depression Inventory; RSES, Rosenberg Self-Esteem Scale; BIS, Barratt Impulsiveness Scale; BMI, Body Mass Index.

**Table 2:** ROI-based between-group comparisons of regional [^11^C]PK11195 BP_ND_ values.

ROIs	[^11^C]PK11195 BP_ND_ Value		*t*-Value	*p*-Value	Effect Size (Cohen’s d)
	MDD Group Mean (SD)	Control Group Mean (SD)			
Lt. PFC	0.245 (0.103)	0.194 (0.104)	1.783	**0.080 ^†^**	0.494
Rt. PFC	0.234 (0.099)	0.192 (0.105)	1.491	0.142	0.413
Lt. ACC	0.268 (0.103)	0.195 (0.104)	2.572	0.013 *	0.713
Rt. ACC	0.157 (0.090)	0.115 (0.103)	1.577	0.121	0.437
Lt. PCC	0.251 (0.122)	0.200 (0.130)	1.467	0.149	0.407
Rt. PCC	0.228 (0.126)	0.142 (0.150)	2.269	0.028 *	0.629
Lt. insula	0.272 (0.114)	0.232 (0.122)	1.240	0.221	0.344
Rt. insula	0.338 (0.125)	0.294 (0.137)	1.221	0.228	0.338
Lt. hippocampus	0.165 (0.088)	0.134 (0.092)	1.222	0.227	0.339
Rt. hippocampus	0.129 (0.086)	0.099 (0.078)	1.347	0.184	0.373
Lt. temporal cortex	0.159 (0.097)	0.124 (0.088)	1.388	0.171	0.385
Rt. temporal cortex	0.170 (0.090)	0.133 (0.082)	1.540	0.130	0.427

Each regional BP_ND_ estimate is the mean determination in MDD patients (*n* = 30) and healthy controls (*n* = 23). * Asterisks indicate statistical significance at *p* < 0.05. ^†^ Cross mark indicates a tendency at *p* < 0.1. ROI, region of interest; BP_ND_, binding potential with respect to non-displaceable compartment; MDD, major depressive disorder; SD, standard deviation; Lt, Left; Rt, Right; PFC, prefrontal cortex; ACC, anterior cingulate cortex; PCC, posterior cingulate cortex.

**Table 3:** Correlation coefficients between regional [^11^C]PK11195 BP_ND_ values and clinical scores for the entire subject group (*n* = 53).

ROIs	RSES		BIS	
	*r*-Value	*p*-Value	*r*-Value	*p*-Value
Lt. prefrontal cortex	−0.242	**0.081 ^†^**	0.114	0.416
Rt. prefrontal cortex	−0.220	0.114	0.098	0.484
Lt. anterior cingulate cortex	−0.301	0.029 *	0.165	0.238
Rt. anterior cingulate cortex	−0.248	**0.074 ^†^**	0.159	0.255
Lt. posterior cingulate cortex	−0.203	0.144	0.163	0.244
Rt. posterior cingulate cortex	−0.269	**0.052 ^†^**	0.198	0.155
Lt. insula	−0.220	0.113	0.144	0.303
Rt. Insula	−0.206	0.139	0.105	0.455
Lt. hippocampus	−0.149	0.285	0.099	0.479
Rt. hippocampus	−0.126	0.370	0.175	0.211
Lt. temporal cortex	−0.217	0.119	0.111	0.429
Rt. temporal cortex	−0.212	0.127	0.106	0.448

* Asterisk indicates statistical significance at *p* < 0.05. ^†^ Cross marks indicate the tendency at *p* < 0.1. BP_ND_, binding potential with respect to non-displaceable compartment; ROI, region of interest; PFC, prefrontal cortex; RSES, Rosenberg Self-Esteem Scale; BIS, Barratt Impulsiveness Scale; Lt, Left; Rt, Right.

**Table 4:** Correlation coefficients between regional [^11^C]PK11195 BP_ND_ values and serum adiponectin levels with BMI as a covariate and between-group comparisons of the correlation coefficients.

ROIs	MDD Group (*n* = 26)	Control Group (*n* = 21)	*z*-Value	*p*-Value
	*r*-Value (*p*-Value)	*r*-Value (*p*-Value)		
Lt. prefrontal cortex	−0.328 (0.109)	0.319 (0.170)	−2.134	0.033 *
Rt. prefrontal cortex	−0.372 (0.067)	0.330 (0.156)	−2.330	0.020 *
Lt. anterior cingulate cortex	−0.294 (0.154)	0.403 (0.078)	−2.320	0.020 *
Rt. anterior cingulate cortex	−0.368 (0.070)	0.348 (0.133)	−2.381	0.017 *
Lt. posterior cingulate cortex	−0.184 (0.378)	0.136 (0.568)	−1.026	0.305
Rt. posterior cingulate cortex	−0.152 (0.468)	0.111 (0.643)	−0.839	0.401
Lt. insula	−0.240 (0.247)	0.195 (0.409)	−1.408	0.159
Rt. Insula	−0.297 (0.149)	0.278 (0.236)	−1.879	0.060
Lt. hippocampus	−0.433 (**0.030**) *	0.520 (**0.019**) *	−3.305	0.001 **
Rt. hippocampus	−0.564 (**0.003**) **	0.594 (**0.006**) **	−4.200	<0.001 **
Lt. temporal cortex	−0.298 (0.148)	0.382 (0.096)	−2.256	0.024 *
Rt. temporal cortex	−0.337 (0.100)	0.380 (0.099)	−2.384	0.017 *

Asterisks indicate statistical significance at *p* < 0.05 * and *p* < 0.01 **. BP_ND_, binding potential with respect to non-displaceable compartment; BMI, body mass index; ROI, region of interest; Lt, Left; Rt, Right.

## Data Availability

The data presented in this study are available upon reasonable request from the corresponding author.

## Supplementary Materials

The following are available online at https://www.mdpi.com/article/10.3390/biomedicines10010034/s1, Table S1: Correlation coefficients between [^11^C]PK11195 BP_ND_ values and clinical scores in the MDD group.
