# Supplementary material for: In Vivo Cerebral Translocator Protein (TSPO) Binding and Its Relationship with Blood Adiponectin Levels in Treatment-Naïve Young Adults with Major Depression: A [11C]PK11195 PET Study
Source: Biomedicines. 2021 Dec 24;10(1):34. doi: 10.3390/biomedicines10010034 (PMC8773340; doi:10.3390/biomedicines10010034)
Supplement: Supplementary file 1 [file biomedicines-10-00034-s001.zip › biomedicines-1439349-supplementary.pdf]

Supplementary Material (Table S1)

Supplementary Table S1. Correlation coefficients between [^{11}C]PK11195 BP_{ND} values and clinical scores in the MDD group (n = 30).

ROIs	HAMD-17	BDI	BIS	RSES
Lt. PFC	0.121 (0.524)	0.143 (0.452)	-0.050 (0.794)	-0.091 (0.634)
Rt. PFC	0.105 (0.582)	0.137 (0.469)	-0.020 (0.915)	-0.118 (0.535)
Lt. ACC	0.034 (0.858)	0.085 (0.655)	-0.032 (0.867)	-0.063 (0.741)
Rt. ACC	0.187 (0.323)	0.107 (0.574)	0.074 (0.698)	-0.202 (0.284)
Lt. PCC	0.234 (0.214)	0.156 (0.409)	0.087 (0.648)	-0.058 (0.760)
Rt. PCC	0.190 (0.314)	-0.051 (0.787)	0.143 (0.451)	-0.098 (0.605)
Lt. insula	0.086 (0.651)	0.081 (0.669)	0.022 (0.908)	-0.177 (0.350)
Rt. insula	0.071 (0.709)	0.164 (0.387)	0.036 (0.851)	-0.181 (0.338)
Lt. hippocampus	0.215 (0.254)	0.193 (0.308)	0.036 (0.852)	-0.020 (0.916)
Rt. hippocampus	0.159 (0.401)	0.151 (0.426)	0.145 (0.444)	0.129 (0.498)
Lt. temporal cortex	0.168 (0.374)	0.166 (0.380)	0.063 (0.741)	-0.138 (0.467)
Rt. temporal cortex	0.181 (0.339)	0.172 (0.364)	0.058 (0.762)	-0.092 (0.628)

The numbers in parentheses indicate the p-values. BP_{ND}, binding potential with respect to non-displaceable compartment; MDD, major depressive disorder; ROI, region of interest; HAMD-17, Hamilton Rating Scale for Depression; BDI, Beck Depression Inventory; BIS, Barratt Impulsiveness Scale; RSES, Rosenberg Self-Esteem Scale; Lt, Left; Rt, Right; PFC, prefrontal cortex; ACC, anterior cingulate cortex; PCC, posterior cingulate cortex.
